# Influence of Single-Nucleotide Polymorphisms on Vitamin D Receptor Expression in Periodontal Ligament Fibroblasts as a Response to Orthodontic Compression

**DOI:** 10.3390/ijms232415948

**Published:** 2022-12-15

**Authors:** Erika Calvano Küchler, Agnes Schröder, Gerrit Spanier, Geraldo Thedei, Maria Beatriz Carvalho Ribeiro de Oliveira, Maria Angélica Hueb de Menezes-Oliveira, Peter Proff, Christian Kirschneck

**Affiliations:** 1 Department of Orthodontics, University of Regensburg, 93047 Regensburg, Germany; 2 Department of Biomaterials, University of Uberaba, Uberaba 38010-200, Brazil; 3 Department of Maxillofacial Surgery, University of Regensburg, 93047 Regensburg, Germany

**Keywords:** gene, dental treatment, fibroblast, vitamin D, orthodontics, polymorphisms, tooth movement

## Abstract

This study aimed to evaluate if single-nucleotide polymorphisms (SNPs) in the vitamin D receptor (VDR) gene are associated with gene expression in human periodontal ligament (hPDL) fibroblasts under simulated orthodontic compressive force. hPDL samples from 57 patients were used. A physiological compressive strain was performed to simulate orthodontic tooth movement in pressure areas under cell culture conditions. The RNA from hPDL fibroblasts was isolated to determine the relative gene expression (mRNA) of the *VDR*. The DNA was also isolated for the genotyping analysis of five SNPs in the *VDR* gene: BglI (rs739837, G/T), BsmI (rs1544410, T/C), ApaI (rs7975232, A/C), FokI (rs2228570, A/G), and TaqI (rs731236, A/G). Real-time polymerase chain reaction was used for both analyses. Kruskal–Wallis tests were used to compare *VDR* expression among genotypes of each SNP. A linear regression analysis was performed to evaluate SNP–SNP interaction. An established alpha of 5% was used. The relative mRNA *VDR* expression according to the genotypes in the SNPs BglI, BsmI, ApaI, FokI, and TaqI was not statistically significantly different (*p* > 0.05). The SNP–SNP interaction evaluated by regression analysis did not demonstrate any statistically significant association. No association was observed (*p* > 0.05). In conclusion, the SNPs BglI (rs739837), BsmI (rs1544410), ApaI (rs7975232), FokI (rs2228570), and TaqI (rs731236) did not show an impact on *VDR* gene expression in hPDL fibroblasts under simulated orthodontic compressive force.

## 1. Introduction

The periodontal ligament (PDL) is a connective tissue inserted into the root cementum on one side and into the alveolar bone on the other side of the periodontal gap that plays an integral part in maintaining periodontal tissue homeostasis. PDL cells are fibroblast-like cells characterized by collagen production but also possess some osteoblastic features [1]. Orthodontic tooth movement (OTM) involves the application of a mechanical force to a tooth by orthodontic appliances, forming tensile and pressure zones in the PDL [2]. OTM consequently leads to the participation of PDL cells in alveolar bone remodeling [3] but does not affect the periodontal and microbiological parameters [4]. Due to the numerous effects of vitamin D3, its supplementation has been targeted as a potential adjuvant therapeutic approach for orthodontic treatment. Several in vitro and in vivo studies have investigated the role of vitamin D on OTM in the past decades [5], suggesting that vitamin D can enhance tooth movement [6,7] and also increase the stability of tooth position after orthodontic treatment, preventing relapse [8].

Vitamin D3 binds to its intracellular receptor to exert its functions; this receptor is called the vitamin D receptor (VDR). The VDR is a member of the nuclear receptor superfamily of transcription factors. The VDR is well known due the fact that it mediates the biological effects of vitamin D3 [9]. The gene that encodes the VDR protein is located on chromosome 12 in humans, and has five promoters, eight coding exons, and six untranslated exons [10]. It is well known that the *VDR* presents many polymorphic regions [11,12] such as single-nucleotide polymorphisms (SNPs). SNPs are DNA sequence variations that occur when a single nucleotide differs among members of a biological species or paired chromosomes in an individual. SNPs can influence the expression and/or functions of the *VDR* and have been explored in complex traits, including oral phenotypes, such as periodontal disease [13,14,15], external apical root resorption as a sequela of orthodontic treatment [16], developmental dental alterations [17,18,19,20,21], and malocclusion [22]. Some recent studies have also shown that vitamin D3 has an effect on the PDL. The studies also demonstrate that PDL cells express the *VDR* gene [23,24,25,26,27].

Additionally, our previous study [23] suggested that functional differences between genotypes in some SNPs in the *VDR* gene may affect mRNA expression of the VDR in hPDL fibroblasts under simulated OTM conditions in vitro. Therefore, in the present study, we decided to replicate our previous study in a larger sample and also to explore new SNPs in the *VDR* in order to unravel the role of genetic polymorphisms on *VDR* expression in periodontal fibroblasts during simulated orthodontic compressive force.

## 2. Results

Table 1 shows the genotyping success rate of each SNP; all of them were higher than 90%.

The relative mRNA *VDR* expression results according to the genotypes of the five studied SNPs—BglI (rs739837, G/T), BsmI (rs1544410, T/C), ApaI (rs7975232, A/C), FokI (rs2228570, A/G), and TaqI (rs731236, A/G)—in the *VDR* gene are shown in Table 2. *VDR* expression was not statistically significant different according to the genotypes. An association was not observed (*p* > 0.05).

The SNP–SNP interaction was evaluated through linear regression analysis. The regression analysis did not demonstrate any statistically significant difference. No association was observed (*p* > 0.05). These results are demonstrated in Table 3.

## 3. Discussion

OTM is a process in which the application of a force induces bone resorption (by osteoclasts) on the pressure side and bone apposition (new bone formation by osteoblasts) on the tension side of the periodontal ligament. Thus, the alveolar bone remodeling that follows the application of orthodontic forces is a well-coordinated process resulting from biological cascades of resorption and apposition caused by mechanical forces [28]. In the past years, several research groups have explored the factors involved in OTM in vivo, in vitro, in clinical studies, and in systematic reviews [29,30,31,32]. There are also research groups investigating oral microbiota [4] and natural substances in oral health [33].

It is well known that PDL fibroblasts react to the application of continuous mechanical orthodontic pressure to move teeth, with alterations in the expression of many important genes [32,34,35]. To mediate the activities of vitamin D, the nuclear transcription factor VDR binds sites in the DNA, stimulating the physiological regulation of several genes [36], including OTM-related genes. It is also known that the VDR is expressed in PDL cells [23,24,25,26,27]; thus, the influence of SNPs on the biologic activity of the *VDR* is worth studying. Therefore, in the present study we decided to explore the role of SNPs in different regions of the *VDR* gene on the mRNA expression of the VDR during simulated orthodontic comprehensive forces.

The human PDL cells used in our study are from healthy teeth of biologically unrelated individuals. The cells were obtained by allowing cells to grow out of periodontal ligament tissue from permanent teeth explants kept under growth-stimulating conditions [1]. This protocol has been used in numerous studies that evaluate OTM in vitro [23,32,34,35].

Multiple SNPs of the human *VDR* gene have been identified [37] and investigated in several conditions and phenotypes so far. In our study, we investigated five SNPs that were selected due to their minor allele frequency and potential relevance. It is important that this could be a limitation as we did not cover all the SNPs in different genes. rs2228570 (FokI) is the only missense SNP of the *VDR*. It is located in exon 2 and its base change generates a new initiation codon [25]. It is the only SNP that causes an actual difference in the structure of the VDR protein (methionine-to-threonine substitution) [38]. The influence of rs2228570 on transcriptional activation by the *VDR* is controversial. Some authors observed distinct functional differences between genotypes in rs2228570 [10,37,39], while others did not find differences in transcriptional activity among genotypes [40], including the study by Liu et al. [25], who did not observe a significant difference in human gingival fibroblasts and PDL cells, which is similar to our results. However, Liu et al. [26] observed that the homozygous GG genotype enhances *RANKL* expression and the *RANKL/OPG* ratio in human gingival fibroblasts and periodontal ligament cells, which could connect this SNP with differences in OTM in clinical practice. The *RANKL/OPG* ratio changes in the PDL under OTM pressure conditions [23,30,31,32]. PDL cells supplemented with vitamin D3 under simulated OTM conditions also downregulated the *RANKL/OPG* ratio [23].

The SNP rs731236 (TaqI) is also in the coding region of the *VDR*. The TaqI polymorphism is characterized by a single base transition that leads to a synonymous change at codon 352 in exon 9. Although this is a synonymous SNP, which does not cause a change in amino acid, we selected this SNP due its previous associations observed in the literature, including with periodontitis [15], attachment loss due to periodontal disease [13], and external apical root resorption during orthodontic treatment [16]. TaqI was also associated with increased transcriptional activity and a high serum level of vitamin D3 [12].

We also evaluated the SNP rs739837 (BgII), which is located in the 3′UTR of the *VDR* gene. This SNP was associated with a variety of conditions and phenotypes (NCBI), including periodontal disease [14] and molar incisor hypomineralization [17], and it was also suggested that it could be associated with *VDR* mRNA expression in PDL cells [23]. In the present study with a larger sample, we were not able to confirm that rs739837 is involved in *VDR* mRNA expression. The intronic SNP Apal (rs7975232) also presented a borderline association with *VDR* expression in the previous study [23], but this association was also not confirmed here. The other intronic SNP (BsmI, rs1544410) evaluated here was previously associated with clinical attachment loss due to periodontal disease [13]; however, in the present study, there was no association with *VDR* mRNA expression.

Although in the previous study the SNPs Apal and BgII were associated with *VDR* mRNA expression, it is important to emphasize that the previous study included samples of only nine patients in the genotyping analysis; it is possible that this exploratory investigation led to a type 1 error. In the current study, we evaluated samples from 57 patients. This larger sample presented an SNP frequency similar to that observed in the general population.

The increasing knowledge of the genetic factors affecting the biology of the orthodontic patient may improve the predictability and control of the direction and speed of OTM. To date, little research has been dedicated to exploring genetic factors that could influence gene expression during OTM. The identification of SNPs that can impact orthodontic treatment should receive attention from researchers.

## 4. Materials and Methods

### 4.1. Ethics Statement

The study protocol was approved by the institutional review board of the University of Regensburg, Germany (12-170-0150), and written informed consent was obtained from each participant in accordance with the Helsinki Declaration of 1975, as revised in 2013.

### 4.2. Sample Collection and In Vitro Compression Model for Orthodontic Tooth Movement

We collected human periodontal ligament (hPDL) from permanent teeth that were caries-free and without restorations. All teeth were extracted in a routine dental treatment at the maxillofacial surgery clinic at the University of Regensburg. If the patient had more than one tooth extracted, only one tooth per patient was selected.

**Figure 1:**
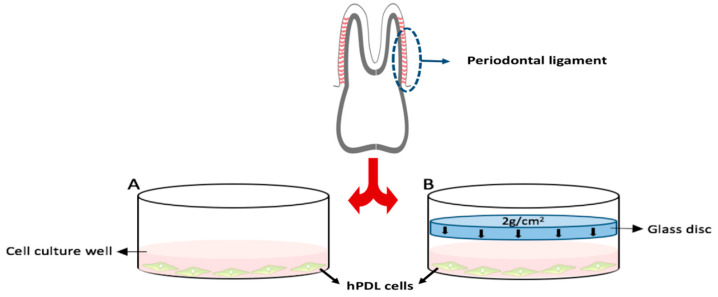
Experimental design. A—Physiological control (no pressure). B—Simulated orthodontic compressive force of 2 g/cm^2^ applied by a 17.1 g glass disc (right side).

A total of 57 hPDL samples (1 per patient) was used. The sample size was determined based on our previous study [23] to detect an expected mean difference of 0.7 with a power of 80% and a significance level of 5%, assuming a standard deviation of 0.6. The hPDL was collected, isolated, cultivated, and characterized according to a well-established protocol, previously published [22,34,35]. Briefly, tissue from hPDL samples were grown in 6-well cell culture plates until proliferation of adherently growing hPDL fibroblasts under normal cell culture conditions (37 °C, 5% CO_2_, water-saturated) in full media. The media consisted of DMEM high glucose (D5796, Sigma-Aldrich^®^, Munich, Germany), 10% FCS (P30–3306, PAN-Biotech, Aidenbach, Germany), 1% L-glutamine (SH30034.01, GE-Healthcare-Europe, Munich, Germany), 100 µM ascorbic acid (A8960, Sigma-Aldrich^®^, St. Louis, MO, USA), and 1% antibiotics/antimycotics (A5955, Sigma-Aldrich^®^). Fibroblasts were identified through their morphological spindle shape and the hPDL-specific marker gene, as previously reported and described in Kirschneck et al. in 2017 [34]. They were frozen in liquid nitrogen (90% FCS, 10% DMSO, freezing 1 °C/min) until use for further studies. For the cell culture experiments, pooled hPDL fibroblasts of the fifth passage were randomly seeded at a density of 2000 cells per mm^2^ onto 6-well cell culture plates. A physiological compressive strain (orthodontic force) of 2 g/cm^2^ using a sterile glass disc was applied for 48 h to simulate orthodontic tooth movement in pressure areas of the hPDL. This was performed under cell culture conditions at 70% confluency without changing the cell culture medium, according to published protocols [22,34,35]. Figure 1 shows the scheme of the in vitro experiment.

### 4.3. Total RNA Isolation and Quantification of Relative Gene Expression (RT-qPCR)

The RNA from hPDL fibroblasts was isolated using 1 mL peqGOLD TriFast^TM^ (PEQLAB Biotechnology GmbH, Erlangen, Germany) following the instructions of the manufacturer. Total RNA was instantaneously cooled on ice. Total RNA amount and purity were photometrically measured at 280 nm, 260 nm, and 230 nm (NanoPhotometer N60, Implen, Munich, Germany). For the synthesis of the complementary DNA (cDNA), 500 ng of RNA per sample were transcribed with a Mastercycler^®^ ep realplex-S thermocycler (Eppendorf AG, Hamburg, Germany).

To determine the relative expression of the VDR, a primer mix containing SYBR^®^ Green JumpStart™ Taq ReadyMix™ (S4438, Sigma–Aldrich), the primer pairs, and 5.25 μL nuclease-free H_2_O (T143, Carl-Roth GmbH) were mixed with 1.5 μL cDNA per sample and a 96-well plate was used. RT-qPCR was performed in a realplex master cycler (Eppendorf AG, Hamburg, Germany). The VDR forward primer (5′-3′) was TAAGACCTACGACCCCA, and the reverse primer (5′-3′) was CTGGGAGTGTGTCTGGAGTTG. For normalization of the target gene, two previously established reference genes that are stable in hPDL fibroblasts were used [34]: RPL22 [forward primer (5′-3′) was TGATTGCACCCACCCTGTAG, and the reverse primer (5′-3′) was GGTTCCCAGCTTTTCCGTTC]; and PPIB [forward primer (5′-3′) was TGATTGCACCCACCCTGTAG, and the reverse primer (5′-3′) was GGTTCCCAGCTTTTCCGTTC]. The primers were designed according to MIQE quality guidelines using NCBI Primer-BLAST and additional software. Eurofins MWG Operon LLC (Huntsville, AL, USA; High Purity Salt Free Purification HPSF^®^) synthesized and purified the primers. A non-template control without cDNA was tested to evaluate primer dimers or contaminating DNA. The relative VDR expression was calculated as follows: 2^−ΔCq^ with ΔCq = Cq (VDR) − Cq (mean RPL22/PPIB), divided by the respective arithmetic 2^−ΔCq^ mean of the untreated samples.

### 4.4. DNA Extraction and Genotyping Analysis

The DNA of hPDL cell samples was isolated using the GenElute Mammalian Genomic DNA Miniprep kit (Sigma Aldrich, Munich, Germany). DNA extraction was performed according to the manufacturer’s instructions. For purity and quantification of the DNA, optical density was measured at 260 nm and 230 nm (NanoPhotometer N60, Implen, Munich, Germany). The OD_260nm/280nm_ ratio > 1.8 indicated protein-free DNA.

A total of five SNPs in the VDR gene, which is located in 12q13.11, were selected based on their minor allele frequency (>10%) and their previously reported association. The SNPs’ characteristics are presented in Table 1. Genotyping was performed by real-time PCR using the TaqMan assay in the Mastercycler^®^ ep realplex-S thermocycler (Eppendorf AG, Hamburg, Germany). The SNPs were blindly genotyped using TaqMan assay. PCR reactions were performed in a total final volume of 5 μL (2.5 μL Taqman genotyping master mix, 0.125 μL SNP assay; Applied Biosystems, Foster City, CA, and 4 ng DNA/reaction in water). Thermal cycling was performed by starting with a hold cycle of 95 °C for 10 min, followed by 40 amplification cycles of 92 °C for 15 s and 60 °C for 1 min.

### 4.5. Statistical Analysis

The software GraphPad Prism 8.0.1 (GraphPad Software Inc., San Diego, CA, USA) was used for statistical analyses. The Shapiro–Wilk test was used to assess the normality of the gene expression data and Levene’s test to determine homogeneity of variance across groups. A non-parametric Kruskal–Wallis test was used to evaluate significant differences between three or more groups. We used Kruskal–Wallis with Dunn’s multiple comparison test to compare the levels of VDR expression among the three genotypes of each SNP in the co-dominant model. The post hoc tests used were Dunn’s tests. A linear regression analysis was performed to evaluate SNP–SNP interaction. Statistical significance was established at 5% for all analyses (*p* < 0.05).

## 5. Conclusions

The SNPs BglI (rs739837), BsmI (rs1544410), ApaI (rs7975232), FokI (rs2228570), and TaqI (rs731236) did not show an impact on *VDR* gene expression in hPDL fibroblasts during simulated orthodontic compressive force. Further studies with larger samples and other SNPs in candidate genes are needed.

## Figures and Tables

**Table 1:** Characteristics of the SNPs and genotyping success rate of each SNP.

SNP	Reference Sequence	^#^ Base Change	^#^ Gene Function and Location	* Genotyping Rate
BgII	rs739837	G/T	3 Prime UTR Variant	54/57 (94.6%)
BsmI	rs1544410	T/C	Intron Variant	55/57 (96.5%)
Apal	rs7975232	A/C	Intron Variant	57/57 (100%)
FokI	rs2228570	A/G	Missense Variant	57/57 (100%)
TaqI	rs731236	A/G	Synonymous Variant	53/57 (92.9%)

Note: * means PCR success amplification rate. ^#^ https://www.ncbi.nlm.nih.gov/snp, accessed on 5 May 2022.

**Table 2:** VDR expression according to the genotypes.

SNP	Genotype	n	Fold Change *VDR* Expression			*p*-Value
			Median	25th Percentile	75th Percentile	
BgII	GG	12	0.78	0.36	1.18	0.874
	GT	27	0.74	0.52	1.16	
	TT	15	0.95	0.48	1.65	
BsmI	CC	17	0.97	0.36	1.61	0.579
	CT	34	0.68	0.54	1.12	
	TT	5	1.01	0.94	1.41	
Apal	AA	14	0.84	0.54	1.42	0.844
	AC	27	0.87	0.43	1.62	
	CC	16	0.65	0.45	1.17	
FokI	AA	8	0.69	0.57	1.18	0.938
	AG	25	0.87	0.41	1.22	
	GG	24	0.89	0.50	1.20	
TaqI	AA	4	0.87	0.66	1.30	0.901
	AG	39	0.80	0.48	1.97	
	GG	12	0.71	0.42	1.22	

Note: Kruskal–Wallis test was used. No statistically significant differences were observed (*p* > 0.05).

**Table 3:** Linear regression analysis to evaluate SNP–SNP interaction.

Variable	Reference *	Estimate	Standard Error	95% CI	t
BgII [GT]	GG	−1.33	1.50	−4.37 to 1.71	0.88
BgII [TT]		−1.40	1.71	−4.85 to 2.05	0.81
BsmI [CT]	CC	−1.32	1.09	−3.54 to 0.89	1.20
BsmI [TT]		0.36	2.05	−3.77 to 4.51	0.17
Apal [AC]	CC	0.18	1.25	−2.36 to 2.72	0.14
Apal [AA]		0.56	1.66	−2.79 to 3.92	0.33
FokI [AA]	AG	0.01	1.27	−2.55 to 2.59	0.01
FokI [GG]		0.67	1.03	−1.41 to 2.76	0.65
TaqI [GG]	AA	0.19	2.30	−4.45 to 4.84	0.08
TaqI [AG]		1.55	2.10	−2.70 to 5.80	0.73

Note: * genotypes used as a reference in the analysis. No statistically significant differences were observed (*p* > 0.05).

## Data Availability

Data are contained within the article or the supplementary material.

## Supplementary Materials

The following supporting information can be downloaded at: https://www.mdpi.com/article/10.3390/ijms232415948/s1.
